# Trends in Chronic Kidney Disease Care in the US by Race and Ethnicity, 2012-2019

**DOI:** 10.1001/jamanetworkopen.2021.27014

**Published:** 2021-09-27

**Authors:** Chi D. Chu, Neil R. Powe, Charles E. McCulloch, Deidra C. Crews, Yun Han, Jennifer L. Bragg-Gresham, Rajiv Saran, Alain Koyama, Nilka R. Burrows, Delphine S. Tuot

**Affiliations:** 1 Division of Nephrology, University of California, San Francisco; 2 OptumLabs Visiting Fellow, OptumLabs, Eden Prairie, Minnesota; 3 Department of Medicine, University of California, San Francisco; 4 Department of Medicine, Mark Zuckerberg and Priscilla Chan San Francisco General Hospital, San Francisco, California; 5 Center for Vulnerable Populations, University of California, San Francisco; 6 Department of Epidemiology and Biostatistics, University of California, San Francisco; 7 Division of Nephrology, Johns Hopkins University School of Medicine, Baltimore, Maryland; 8 Johns Hopkins Center for Health Equity, Johns Hopkins Medical Institutions, Baltimore, Maryland; 9 Division of Nephrology, Department of Medicine, University of Michigan, Ann Arbor; 10 Kidney Epidemiology and Cost Center, University of Michigan, Ann Arbor; 11 Division of Diabetes Translation, Centers for Disease Control and Prevention, Atlanta, Georgia

## Abstract

**Importance:**

Significant racial and ethnic disparities in chronic kidney disease (CKD) progression and outcomes are well documented, as is low use of guideline-recommended CKD care.

**Objective:**

To examine guideline-recommended CKD care delivery by race and ethnicity in a large, diverse population.

**Design, Setting, and Participants:**

In this serial cross-sectional study, adult patients with CKD that did not require dialysis, defined as a persistent estimated glomerular filtration rate less than 60 mL/min/1.73 m^2^ or a urine albumin-creatinine ratio of 30 mg/g or higher for at least 90 days, were identified in 2-year cross-sections from January 1, 2012, to December 31, 2019. Data from the OptumLabs Data Warehouse, a national data set of administrative and electronic health record data for commercially insured and Medicare Advantage patients, were used.

**Exposures:**

The independent variables were race and ethnicity, as reported in linked electronic health records.

**Main Outcomes and Measures:**

On the basis of guideline-recommended CKD care, the study examined care delivery process measures (angiotensin-converting enzyme inhibitor or angiotensin II receptor blocker prescription for albuminuria, statin prescription, albuminuria testing, nephrology care for CKD stage 4 or higher, and avoidance of chronic nonsteroidal anti-inflammatory drug prescription) and care delivery outcome measures (blood pressure and diabetes control).

**Results:**

A total of 452 238 patients met the inclusion criteria (mean [SD] age, 74.0 [10.2] years; 262 089 [58.0%] female; a total of 7573 [1.7%] Asian, 49 970 [11.0%] Black, 15 540 [3.4%] Hispanic, and 379 155 [83.8%] White). Performance on process measures was higher among Asian, Black, and Hispanic patients compared with White patients for angiotensin-converting enzyme inhibitor and angiotensin II receptor blocker use (79.8% for Asian patients, 76.7% for Black patients, and 79.9% for Hispanic patients compared with 72.3% for White patients in 2018-2019), statin use (72.6% for Asian patients, 69.1% for Black patients, and 74.1% for Hispanic patients compared with 61.5% for White patients), nephrology care (64.8% for Asian patients, 72.9% for Black patients, and 69.4% for Hispanic patients compared with 58.3% for White patients), and albuminuria testing (53.9% for Asian patients, 41.0% for Black patients, and 52.6% for Hispanic patients compared with 30.7% for White patients). Achievement of blood pressure control to less than 140/90 mm Hg was similar or lower among Asian (71.8%), Black (63.3%), and Hispanic (69.8%) patients compared with White patients (72.9%). Achievement of diabetes control with hemoglobin A_1c_ less than 7.0% was 50.1% in Asian patients, 49.3% in Black patients, and 46.0% in Hispanic patients compared with 50.3% for White patients.

**Conclusions and Relevance:**

Higher performance on CKD care process measures among Asian, Black, and Hispanic patients suggests that differences in medication prescription and diagnostic testing are unlikely to fully explain known disparities in CKD progression and kidney failure. Improving care delivery processes alone may be inadequate for reducing these disparities.

## Key Points

**Question:**

Are there differences in guideline-recommended care delivery for chronic kidney disease (CKD) by race and ethnicity?

**Findings:**

In this cross-sectional study of 452 238 commercially insured and Medicare Advantage US adults with CKD from 2012 to 2019, compared with White patients, Asian, Black, and Hispanic patients had higher performance across many care delivery measures (eg, statin use and renin-angiotensin blockade) but similar or poorer performance on blood pressure and diabetes control.

**Meaning:**

Higher performance on multiple CKD care measures among Asian, Black, and Hispanic patients suggests that differences in care delivery are unlikely to fully explain known disparities in CKD progression and kidney failure.

## Teaser

This cross-sectional study evaluates trends in chronic kidney disease care delivery by race and ethnicity in a large population of US adults with chronic kidney disease.

## Introduction

With a steady prevalence of 13% to 15% for nearly 2 decades in the adult US population,^1^ chronic kidney disease (CKD) is a major public health burden and an important cause of morbidity and mortality.^2^ Timely identification and use of effective, evidence-based therapies are critical for preventing CKD progression and the associated risks of end-stage kidney disease (ESKD), cardiovascular morbidity, and mortality.^3^

Given racial and ethnic disparities in ESKD and cardiovascular disease, ensuring consistent, evidence-based care delivery is a foundation for achieving health equity.^4^ Racial and ethnic minority populations shoulder a disproportionate burden of CKD and ESKD as well as comorbid risk factors for CKD development and progression, such as diabetes and hypertension.^5^ Faster progression of CKD with several-fold greater ESKD incidence among Black and Hispanic patients compared with White patients has been consistently documented, underscoring the importance of ensuring timely, effective preventive care for CKD among minority populations.^6,7^

Evidence-based clinical practice guidelines from professional societies (eg, Kidney Disease: Improving Global Outcomes [KDIGO], American College of Cardiology/American Heart Association, and American Diabetes Association) provide recommendations across the spectrum of CKD care from detection to risk stratification to delivery of effective treatments, such as blood pressure control and use of angiotensin-converting enzyme inhibitors (ACEis) and angiotensin II receptor blocker (ARB) medications.^8,9,10,11,12,13^ However, published data have consistently suggested major gaps in implementation of numerous components of CKD-related care. Nationally representative survey data indicate that only 36% of US adults with CKD receive a statin^14^ and 25% to 50% receive ACEi or ARB therapy.^15,16^ Among Medicare patients older than 65 years with diagnosed CKD, 40% to 50% had urine albumin testing in accordance with guideline recommendations.^1^ The objective of this study was to evaluate trends in CKD care delivery by race and ethnicity in a large population of US adults with CKD (defined using laboratory criteria) who are actively engaged in medical care.

## Methods

### Study Design

We conducted a serial cross-sectional analysis using data from the OptumLabs Data Warehouse, which includes deidentified medical claims, pharmacy claims, electronic health record (EHR) data, and laboratory results from commercially insured and Medicare Advantage enrollees throughout the US.^17^ Because this study involved analysis of preexisting deidentified data, the University of California, San Francisco Institutional Review Board considered this study exempt from review, and the need for informed consent was waived. This study followed the Strengthening the Reporting of Observational Studies in Epidemiology (STROBE) reporting guideline.^18^

### Study Population

We assembled serial cross-sections that comprise patients with CKD from 4 consecutive periods: 2012 to 2013, 2014 to 2015, 2016 to 2017, and 2018 to 2019. Within each period, we identified adults (≥18 years of age) with at least 1 outpatient encounter during that period. We included patients with CKD, defined according to the KDIGO criteria as those with outpatient laboratory values at least 90 days apart demonstrating persistence of a low eGFR (<60 mL/min/1.73 m^2^) or persistence of albuminuria (urine albumin-creatinine ratio [UACR] ≥30 mg/g).^8^ The CKD-Epidemiology Collaboration equation was used to calculate the eGFR using age, sex, race (Black vs other race), and serum creatinine level.^19^ We included race in the calculation of eGFR in order to most closely capture the eGFR reporting that physicians in the study period would have seen and acted upon. The date of the second laboratory value was defined as the index date for each patient. For patients who met the eGFR and UACR criteria for CKD, the earlier of the 2 was the index date. To ensure adequate ascertainment and minimize potential bias associated with loss to follow-up, we required patients to have at least 365 days of continuous enrollment (medical and pharmacy coverage) before and after the index date. Medical claims, pharmacy claims, and laboratory results were extracted for each included patient. We excluded patients with ESKD before the index date, identified by *Current Procedural Terminology 4* codes for dialysis-related procedures,^20^ as well as those with unknown race or ethnicity.

### Variables

The independent variable was race or ethnicity as recorded in EHR records, which may reflect classification by patients or clinical staff. Race and ethnicity were categorized into Asian, Black, Hispanic, or White; categories were mutually exclusive. Although individual health system EHRs may have included additional options (ie, for American Indian or Alaska Native), we were unable to ascertain these data because these patients were classified as other in the data set; therefore, these patients were not included in our analysis. Blood pressure was extracted from EHR records, using the outpatient blood pressure nearest to the index date for each patient. Hypertension was defined as systolic blood pressure of 140 mm Hg or higher, diastolic blood pressure of 90 mm Hg or higher, or use of antihypertensive medications. Diabetes was defined as a hemoglobin A_1c_ level of 6.5% or higher (to convert to proportion of total hemoglobin, multiply by 0.01) or use of diabetes medications. Pharmacy claims were used to ascertain medication use (eTable 1 in Supplement 1). Other comorbidities, including congestive heart failure, coronary heart disease, and cerebrovascular disease, were identified using *International Classification of Diseases, Ninth Revision (ICD-9)* and *International Statistical Classification of Diseases and Related Health Problems, Tenth Revision (ICD-10)* codes from physician and facility claims (eTable 2 in Supplement 1).

### Outcomes

We examined CKD care delivery process and outcome measures based on KDIGO clinical practice guidelines for CKD. Delivery process measures included (1) ACEi or ARB use among patients with a UACR of 30 mg/g or higher (if diabetes or hypertension was present) or a UACR of 300 mg/g or higher (irrespective of diabetes or hypertension status), (2) statin prescription among patients 50 years or older, (3) avoidance of long-term nonsteroidal anti-inflammatory drug (NSAID) prescription, (4) nephrology care among patients with an eGFR less than 30 mL/min/1.73 m^2^, and (5) annual UACR monitoring. Delivery outcome measures included (1) blood pressure controlled to less than 140/90 mm Hg, (2) blood pressure controlled to less than 130/80 mm Hg, and (3) diabetes control (hemoglobin A_1c_ <7.0%) among patients with diabetes. Detailed specifications and references for each CKD care delivery outcome are provided in Table 1.

**Table 1.** Guideline-Based Performance Metrics Evaluated and Operational Definitions

Metric	Guideline recommendation^a^	Numerator	Denominator
**Process measures**			
ACEi and ARB use in albuminuria	3.1.6: We suggest that an ARB or ACEi be used in adults with diabetes with CKD and urine albumin excretion of 30 to 300 mg/24 h (or equivalent). (2D) 3.1.7: We recommend that an ARB or ACEi be used in adults with and without diabetes with CKD and urine albumin excretion >300 mg/24 h (or equivalent). (1B) 3.4: We suggest that an ARB or ACEi be used in adults without diabetes with CKD and urine albumin excretion of 30-300 mg per 24 h in whom treatment with BP-lowering drugs is indicated. (2D) 3.5: We recommend that an ARB or ACEi be used in adults without diabetes with CKD and urine albumin excretion >300 mg per 24 h in whom treatment with BP-lowering drugs is indicated. (1B)	Patients having at least 1 pharmacy claim for an ACEi or ARB medication within 1 y of the index date	Patients with diabetes or hypertension and UACR nearest the index date that was ≥30 mg/g or with UACR≥300 mg/g irrespective of diabetes or hypertension status
Statin use if ≥50 y of age	2.1.1: In adults ≥50 y of age with an eGFR <60 mL/min/1.73 m^2^ but not treated with chronic dialysis or kidney transplantation (GFR categories G3a-G5), we recommend treatment with a statin or a statin-ezetimibe combination. (1A) 2.1.2: In adults ≥50 y with CKD and an eGFR ≥60 mL/min/1.73 m^2^ (GFR categories G1-G2), we recommend treatment with a statin. (1B)	Patients having at least 1 pharmacy claim for a statin medication within 1 y of the index date	Patients ≥50 y of age
Long-term NSAID avoidance	4.4.1: We recommend that prescribers should take GFR into account when drug dosing. (1A) 4.4: We recommend that NSAIDs be avoided in people with GFR <30 mL/min/1.73 m^2^ We recommend that prolonged therapy with NSAIDs not be used in people with GFR <60 mL/min/1.73 m^2^	Patients having ≥2 pharmacy claims for NSAIDs within 1 y of the index date	Patients whose eGFR nearest to the index date was <60 mL/min/1.73 m^2^
Referral to a nephrologist if eGFR <30 mL/min/1.73 m^2^	5.1.1: We recommend referral to specialist kidney care services for people with CKD in the following circumstances (1B): GFR <30 mL/min/1.73 m^2^ (GFR categories G4-G5)	Patients having at least 1 outpatient encounter with a nephrologist within 1 y of the index date	Patients whose eGFR nearest to the index date was <30 mL/min/1.73 m^2^
UACR testing	2.1.1: Assess GFR and albuminuria at least annually in people with CKD. Assess GFR and albuminuria more often for individuals at higher risk of progression and/or when measurement will impact therapeutic decisions. (not graded)	Patients having at least 1 UACR measured within 1 y after the index date	All included patients
**Outcome measures**			
BP control <140/90 mm Hg or <130/80 mm Hg	3.1.4: We recommend that in adults with and without diabetes with CKD and urine albumin excretion <30 mg/24 h (or equivalent) whose office BP is consistently >140 mm Hg systolic or >90 mm Hg diastolic be treated with BP-lowering drugs to maintain a BP that is consistently ≤140 mm Hg systolic and ≤90 mm Hg diastolic. (1B) 3.1.5: We suggest that in adults with and without diabetes with CKD and with urine albumin excretion of ≥30 mg/24 h (or equivalent) whose office BP is consistently >130 mm Hg systolic or >80 mm Hg diastolic be treated with BP-lowering drugs to maintain a BP that is consistently ≤130 mm Hg systolic and ≤80 mm Hg diastolic. (2D)	Patients whose outpatient BP nearest to the index date was <140 (or <130) mm Hg systolic and <90 (or <80) mm Hg diastolic	Patients who had at least 1 outpatient BP measured at any time within 1 y of the index date
Diabetes control (HbA_1c_<7.0%)	3.1.15: We recommend a target HbA_1c_ level of approximately 7.0% (53 mmol/mol) to prevent or delay progression of the microvascular complications of diabetes, including diabetic kidney disease. (1A)	Patients whose HbA_1c_ level nearest to the index date was <7.0%	Patients with diabetes, defined as having an HbA_1c_ level ≥6.5%, ≥1 pharmacy claim for a diabetes medication, or ≥1 diagnostic code for diabetes within 1 y of the index date

Abbreviations: ACEi, angiotensin-converting enzyme inhibitor; ARB, angiotensin II receptor blocker; BP, blood pressure; CKD, chronic kidney disease; eGFR, estimated glomerular filtration rate; HbA_1c_, hemoglobin A_1c_; KDIGO, Kidney Disease: Improving Global Outcomes; NSAID, nonsteroidal anti-inflammatory drug; UACR, urine albumin-creatinine ratio.

SI conversion: To convert HbA_1c_ to proportion of total hemoglobin, multiply by 0.01.

^a^ Guidelines are from KDIGO clinical practice guidelines for the evaluation and management of CKD, blood pressure, and lipid management in CKD.^8,9,10^

### Statistical Analysis

We calculated descriptive statistics for demographic and clinical characteristics of patients by race and ethnicity and by study period. Characteristics were compared by study period as well as race and ethnicity using χ^2^ or analysis of variance tests as appropriate. For each CKD care delivery outcome, we computed the proportion of patients who met the outcome for each period. Given previously documented differences in ACEi and ARB use and UACR testing between patients with and without diabetes,^16,21^ we performed supplemental analyses examining these outcomes stratified by diabetes status in addition to race and ethnicity. We also examined care delivery stratified by age (<65 or ≥65 years) because inequalities in care delivery may operate differently among those of working age. We used multivariable logistic regression to examine associations (odds ratios and 95% CIs) between race or ethnicity and each care outcome. We examined unadjusted associations and associations adjusted for demographic characteristics (age and sex), then additionally adjusted for clinical characteristics that are potential confounders (hypertension, diabetes, heart failure, coronary heart disease, cerebrovascular disease, and continuous eGFR).

Because our cohort construction required the presence of at least 2 creatinine or UACR tests, differential testing practices by race and ethnicity could lead to biased results. To assess this possibility, we performed a supplementary analysis that examined the number of creatinine and UACR tests obtained by race and ethnicity among patients at risk for CKD (having hypertension, diabetes, or cardiovascular disease), and we did not find substantively different testing patterns by race and ethnicity (eFigure 1 in Supplement 1).

All statistical tests were 2-sided; *P* < .05 was considered statistically significant. Analyses were performed using R software, version 4.0 (R Foundation for Statistical Computing).

## Results

### Study Population

A total of 452 238 patients met the inclusion criteria (mean [SD] age, 74.0 [10.2] years; 262 089 [58.0%] female; 7573 [1.7%] Asian, 49 970 [11.0%] Black, 15 540 [3.4%] Hispanic, and 379 155 [83.8%] White). Derivation of the study population is shown in eFigure 2 in Supplement 1. Demographic and clinical characteristics of patients were largely similar across study periods, with notable differences in mean age, which increased from 72.0 to 75.2 years between 2012-2013 and 2018-2019, and in the proportion with Medicare Advantage insurance, which increased from 75.9% to 88.8% during the same time (eTable 3 in Supplement 1).

Characteristics of patients with CKD by race and ethnicity are given in Table 2. White patients were older (mean [SD] age, 74.5 [10.0] years) compared with Asian (mean [SD] age, 72.9 [11.7] years) patients, Black (mean [SD] age, 72.1 [10.5] years) patients, and Hispanic (mean [SD] age, 70.4 [12.1] years) patients (*P* < .001). Diabetes prevalence was lower among White patients (38.7%) compared with Asian (56.7%), Black (55.5%), and Hispanic (60.5%) patients. Severe albuminuria (UACR ≥300 mg/g) was also less prevalent among White patients (8.4%) compared with Asian (13.3%), Black (13.6%), and Hispanic (13.7%) patients (*P* < .001).

**Table 2.** Study Population Characteristics by Race and Ethnicity^a^

Characteristic	Asian	Black	Hispanic	White
Total	7573 (1.7)	49 970 (11.0)	15 540 (3.4)	379 155 (83.8)
Demographic characteristics				
Age, mean (SD), y	72.9 (11.7)	72.1 (10.5)	70.4 (12.1)	74.5 (10.0)
Female	3608 (47.6)	33 211 (66.5)	8294 (53.4)	216 976 (57.2)
Male	3965 (52.4)	16 759 (33.5)	7246 (46.6)	162 179 (42.8)
Neighborhood education^b^				
Less than high school	731 (9.8)	1821 (3.7)	1386 (9.1)	2722 (0.7)
High school	2670 (35.7)	33 061 (67.5)	8379 (54.9)	180 554 (48.5)
Less than college	3113 (41.6)	12 810 (26.1)	4891 (32.0)	163 308 (43.9)
College or higher	974 (13.0)	1314 (2.7)	610 (4.0)	25 585 (6.9)
Medicare Advantage	6124 (80.9)	43 132 (86.3)	12 109 (77.9)	320 159 (84.4)
Comorbidities				
Hypertension	6493 (85.7)	46 193 (92.4)	13 676 (88.0)	314 612 (83.0)
BP, mean (SD), mm Hg				
Systolic	130 (19)	134 (20)	132 (19)	130 (18)
Diastolic	72 (11)	75 (11)	72 (11)	72 (11)
Diabetes	4293 (56.7)	27 721 (55.5)	9397 (60.5)	146 783 (38.7)
HbA_1c_, mean (SD), %	6.8 (1.2)	7.0 (1.6)	7.1 (2.2)	6.7 (1.5)
eGFR, mean (SD), mL/min/1.73 m^2^	57 (21)	53 (22)	57 (23)	51 (16)
eGFR, mL/min/1.73 m^2^				
≥60	1933 (25.6)	9347 (18.8)	4182 (27.0)	50 208 (13.3)
45-59	3667 (48.5)	23 012 (46.3)	7046 (45.5)	209 059 (55.3)
30-44	1430 (18.9)	11 932 (23.9)	3029 (19.5)	91 957 (24.3)
<30	528 (7.0)	5465 (11.0)	1239 (8.0)	27 163 (7.2)
UACR, median (IQR), mg/g	34 (10-109)	23 (7-98)	26 (8-98)	20 (8-61)
UACR, mg/g				
<30	2148 (46.5)	13 102 (55.9)	4768 (52.6)	81 030 (60.8)
30-299	1855 (40.2)	7155 (30.5)	3055 (33.7)	40 940 (30.7)
≥300	617 (13.3)	3194 (13.6)	1235 (13.7)	11 278 (8.4)
Hyperlipidemia	6750 (89.1)	42 687 (85.4)	13 707 (88.2)	328 050 (86.5)
Congestive heart failure	1041 (13.7)	13 518 (27.1)	3152 (20.3)	87 283 (23.0)
Coronary heart disease	514 (6.8)	5460 (10.9)	1616 (10.4)	44 168 (11.6)
Cerebrovascular disease	943 (12.5)	9277 (18.6)	2345 (15.1)	58 059 (15.3)

Abbreviations: BP, blood pressure; eGFR, estimated glomerular filtration rate; HbA_1c_, hemoglobin A_1c_; IQR, interquartile range; UACR, urine albumin-creatinine ratio.

SI conversion: To convert HbA_1c_ to proportion of total hemoglobin, multiply by 0.01.

^a^ Data are presented as number (percentage) of patients unless otherwise indicated. All comparisons were significant at *P* < .001.

^b^ Median education level achieved among all residents 25 years and older within the specified census block group according to the American Community Survey.

### Prevalence of CKD Care Delivery by Race and Ethnicity

**Figure 1.**
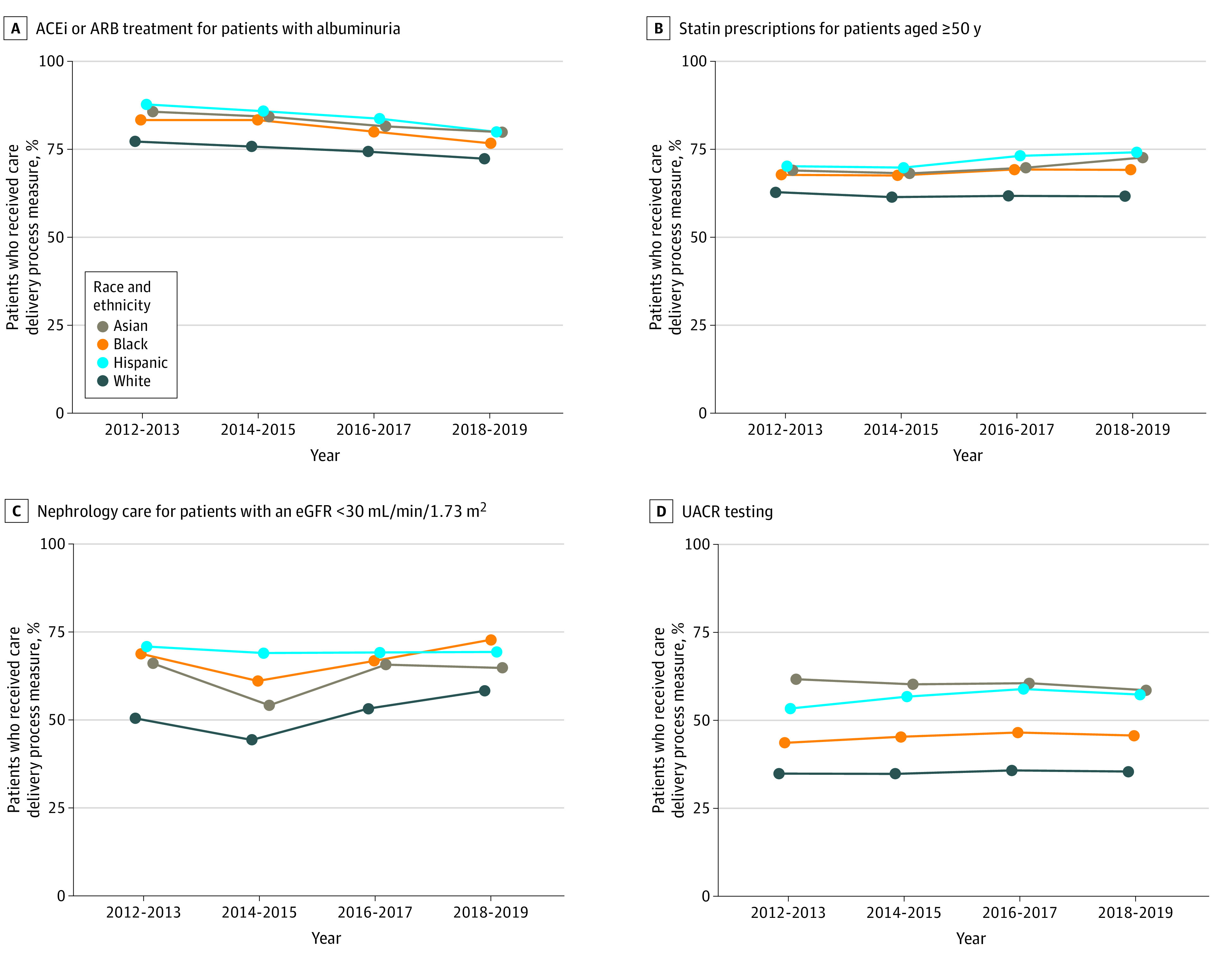
Trends in Chronic Kidney Disease Care Delivery Process Measures by Race and Ethnicity, 2012-2019 ACEi indicates angiotensin-converting enzyme inhibitor; ARB, angiotensin II receptor blocker; eGFR, estimated glomerular filtration rate; UACR, urine albumin-creatinine ratio.

Trends in the number of patients who met each CKD care delivery process measure are shown in Figure 1. No consistent time trends were found except for ACEi and ARB use, which demonstrated a small but consistent decrease over time among all racial and ethnic groups, ranging from 77.2% to 87.7% in 2012-2013 to 72.3% to 79.9% in 2018-2019. For most care delivery process measures, Asian, Black, and Hispanic patients demonstrated greater prevalence of guideline-concordant care compared with their White counterparts. These measures included ACEi and ARB use (79.8% among Asian, 76.7% among Black, and 79.9% among Hispanic patients compared with 72.3% among White patients in 2018-2019), statin use (72.6% among Asian, 69.1% among Black, and 74.1% among Hispanic patients compared with 61.5% among White patients), nephrology care for individuals with an eGFR less than 30 mL/min/1.73 m^2^ (64.8% among Asian, 72.9% among Black, and 69.4% among Hispanic patients compared with 58.3% among White patients), and albuminuria testing (53.9% among Asian, 41.0% among Black, 52.6% among Hispanic patients compared with 30.7% among White patients). Avoidance of long-term prescription NSAID use was consistently greater than 80% across all racial and ethnic groups over time, with modestly lower performance among Black patients (84.9%) and Hispanic patients (82.4%) in 2018-2019 compared with Asian patients (90.1%) and White patients (88.8%).

**Figure 2.**
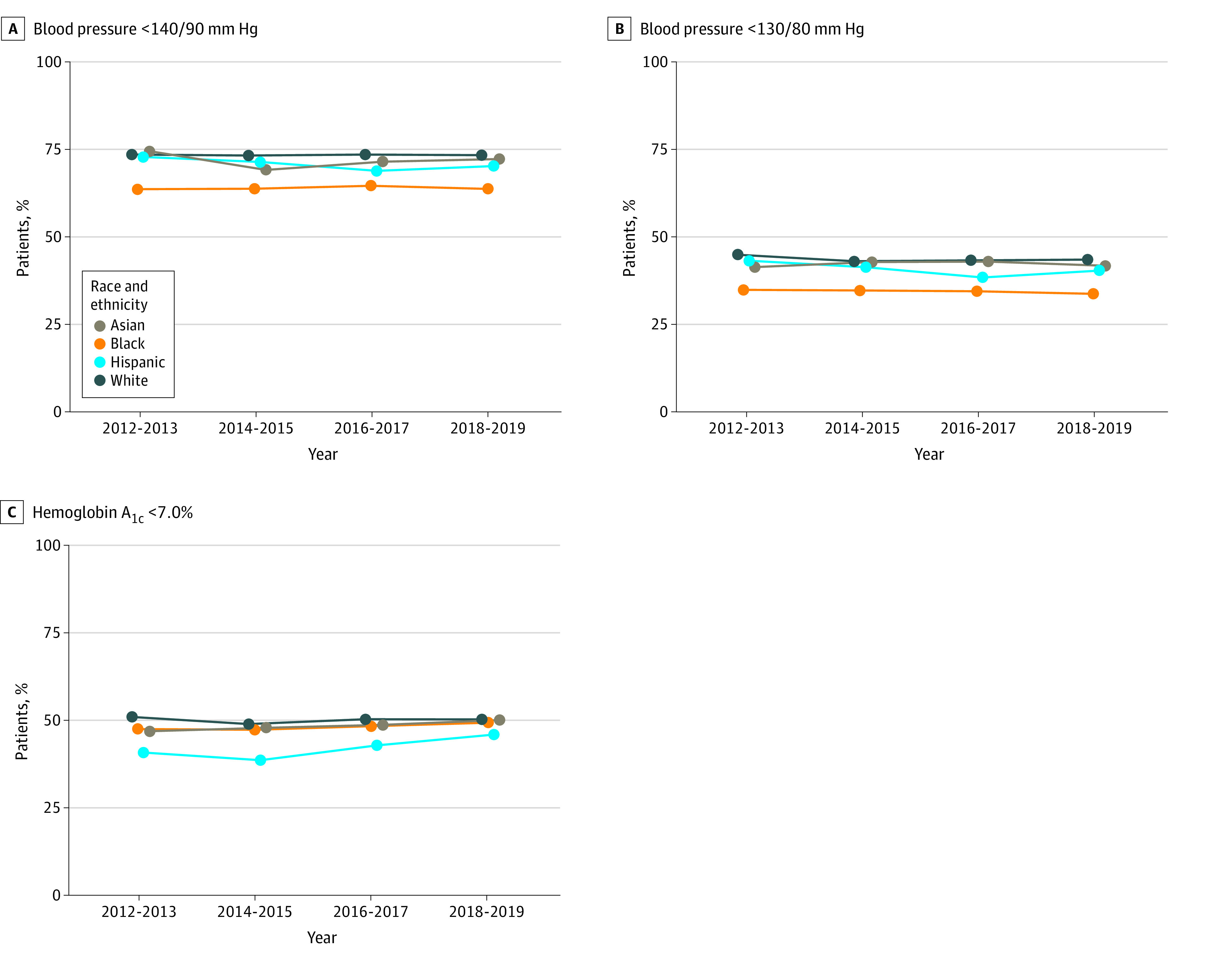
Trends in Blood Pressure and Diabetes Control in Chronic Kidney Disease by Race and Ethnicity, 2012-2019

For care delivery outcome measures (Figure 2), the proportion of patients who met target blood pressures was similar among Asian (41.6%), Hispanic (40.3%), and White (43.4%) patients for the 130/80 mm Hg target and among Asian (71.8%), Hispanic (69.8%), and White (72.9%) patients for the 140/90 mm Hg target in 2018-2019. Black patients had consistently less well-controlled blood pressure, with 33.6% meeting the 130/80 mm Hg target and 63.3% meeting the 140/90 mm Hg target in 2018-2019. The prevalence of hemoglobin A_1c_ less than 7.0% among patients with diabetes was similar among Asian (50.1%), Black (49.3%), and White (50.3%) patients compared with 46.0% among Hispanic patients.

### Prevalence of CKD Care Delivery by Diabetes Status or Age and Race or Ethnicity

When stratified by diabetes status, UACR testing was more than twice as prevalent among patients with diabetes compared with those without (eFigure 3A in Supplement 1). Guideline-concordant ACEi and ARB use was consistently higher among patients with diabetes, with the lowest rates of guideline-concordant ACEi and ARB use among White patients (eFigure 3B in Supplement 1). When results were stratified by age, we found no consistent substantive differences compared with the overall analysis (eFigure 4 in Supplement 1).

### Unadjusted and Adjusted Associations Between Race or Ethnicity and CKD Care Delivery

**Figure 3.**
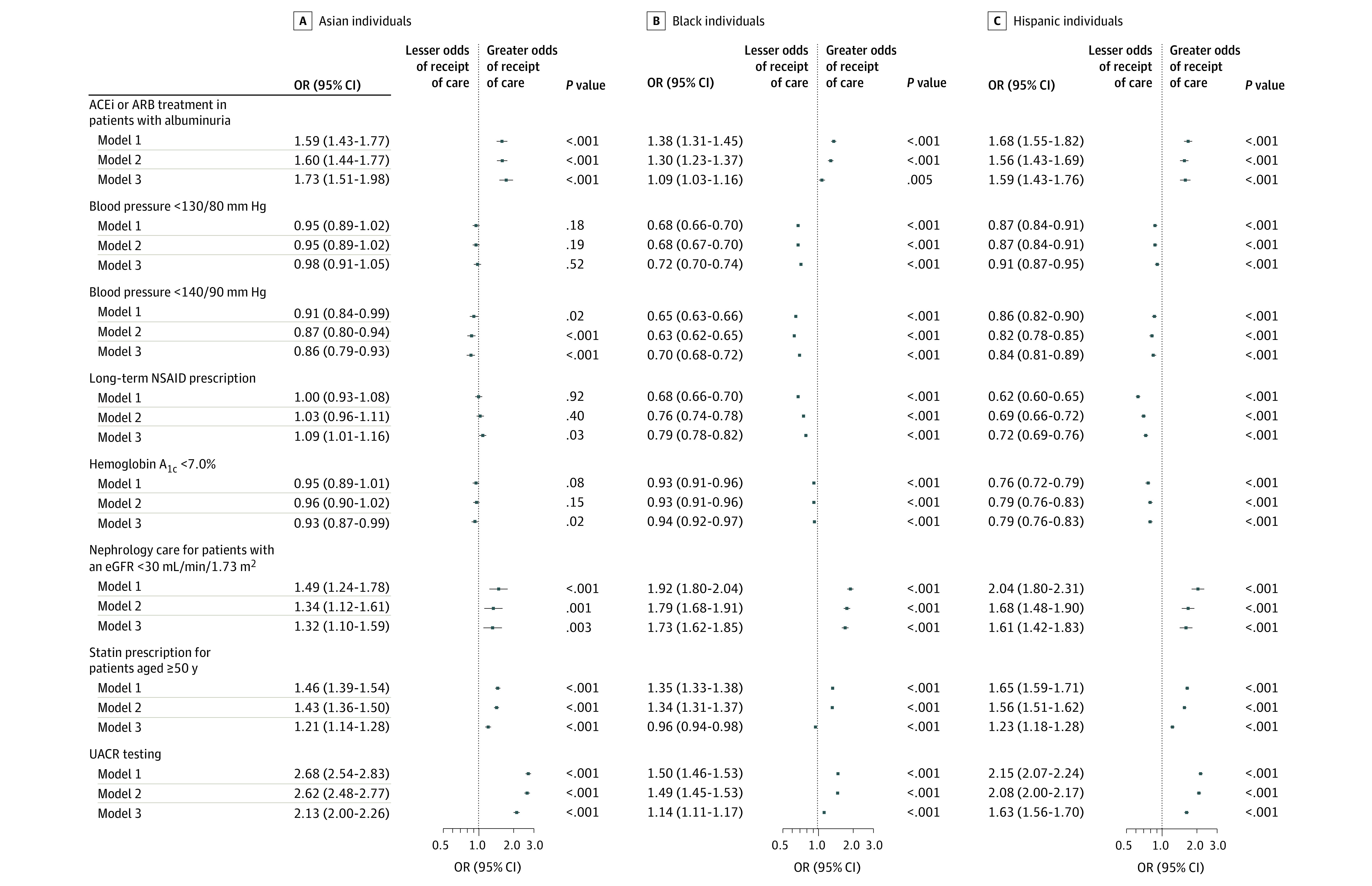
Odds Ratios (ORs) and 95% CIs for Chronic Kidney Disease Care Delivery Outcomes and Race and Ethnicity The reference class for all models is White individuals. Model 1 is unadjusted. Model 2 is adjusted for age and sex. Model 3 is additionally adjusted for hypertension, diabetes (except for hemoglobin A_1c_ outcome), coronary heart disease, cerebrovascular disease, and estimated glomerular filtration rate (eGFR). ACEi indicates angiotensin-converting enzyme inhibitor; ARB, angiotensin II receptor blocker; NSAID, nonsteroidal anti-inflammatory drug; UACR, urine albumin-creatinine ratio.

In multivariable logistic regression, Asian, Black, and Hispanic patients had greater odds of receiving guideline-concordant care for ACEi and ARB use, nephrology care, statin prescription, and UACR testing compared with White patients (Figure 3). Most of these odds ratios were partially attenuated by adjustment for demographic and clinical characteristics, but positive associations persisted (with the exception of statin use among Black patients). For blood pressure and diabetes control, for which Black and Hispanic patients were consistently less likely to meet guideline-recommended targets, negative associations were not substantively changed by multivariable adjustment.

## Discussion

In this cross-sectional study including a large sample of commercially insured and Medicare Advantage patients with CKD, we identified variable adherence to guideline-recommended components of CKD care, including control of blood pressure and diabetes, ACEi or ARB and statin use, nephrology care for advanced CKD, albuminuria testing, and long-term prescription NSAID avoidance. We also found that, compared with White patients, adherence to guideline-recommended care was more likely among Asian, Black, and Hispanic patients for several care delivery process measures, including ACEi and ARB use, statin use, nephrology care, and albuminuria testing, whereas these patients tended to have similar or lower rates of achieving short-term outcomes, such as blood pressure and diabetes control targets. Associations between race or ethnicity and care delivery largely persisted after adjustment for demographic or clinical characteristics.

Given prominent racial and ethnic disparities in CKD,^1^ the higher performance among Asian, Black, and Hispanic patients on multiple care delivery measures, including ACEi or ARB and statin prescription, nephrology care, and UACR testing, was somewhat unexpected. Similar findings have been observed in CKD epidemiologic surveillance data from the Veterans Affairs Health System, where Asian, Hispanic, and non-Hispanic Black veterans were more likely to have filled a prescription for an ACEi or ARB, more likely to see a nephrologist (among patients with an eGFR <30 mL/min/1.73 m^2^), and more likely to have UACR testing than their White counterparts.^22^ Greater guideline-concordant care among patients of other than White races has also been observed in a public safety net health setting and the Military Health System.^21,23^ Reasons for these findings are unclear because they were not fully explained by age, sex, and clinical characteristics in our study or any of the others. Although residual confounding or unmeasured comorbidities remain a possible explanation, we hypothesize that practitioners may be appropriately recognizing increased risk of adverse outcomes (related to CKD or its complications) in minority patient populations, stimulating more UACR testing, referral to a nephrologist, and preventive treatment (statin or ACEi or ARB use).^24,25^ Nevertheless, our results suggest a substantial opportunity for improved CKD care delivery across all racial and ethnic groups, including White patients, who appear to be systematically undertreated across multiple health data sources.

Despite findings of higher performance among Asian, Black, and Hispanic patients for process measures (ACEi and ARB or statin prescription, nephrology care, and UACR testing), we found that racial and ethnic minority populations were similarly or less likely to achieve guideline-recommended targets for blood pressure and diabetes. These findings are consistent with data from general population settings (not limited to CKD), indicating lower prevalence of controlled blood pressure and diabetes among Black and Hispanic populations.^26,27,28,29^ Notably, in the Veterans Administration Health System, both higher guideline-concordant care and faster CKD progression have been observed among Black and Hispanic patients compared with White patients.^30^ Lower achievement of blood pressure and glycemic targets despite better performance on process-type care delivery measures suggests that more aggressive health care—testing, prescribing, and referring to match guideline recommendations—is likely inadequate in isolation for narrowing health disparities. An alternative might be exploring how interventions addressing social determinants of health (eg, food insecurity, housing instability, and health literacy) may help mitigate the burden of CKD risk factors and health consequences among non-White individuals, including Black and Hispanic persons. In addition, genetic risk factors for CKD progression, such as 2 *APOL1* high-risk alleles, are present in 10% to 15% of Black persons and may contribute to persistent racial disparity for Black persons even if health care and other determinants of health are equalized.^31,32^

The flat trajectory of many measures during the past decade suggests a lack of progress across multiple aspects of CKD care. Directing greater attention upstream (ie, toward interventions for optimizing care for CKD and preventing kidney failure) of ESKD may provide the opportunity to prevent the morbidity, mortality, and costs associated with progressive kidney disease.^33^ Notably, given that most patients with CKD have an eGFR of 30 mL/min/1.73 m^2^ or greater with care managed by primary care physicians and not nephrologists, improving evidence-based CKD care delivery will require greater attention to be focused on the primary care setting. A recent evaluation of quality metrics in kidney care revealed that most existing metrics pertained to dialysis care, with a paucity of metrics related to preventing CKD progression and CKD complications.^34^ The need to improve CKD care was also highlighted in the 2019 Advancing American Kidney Health Executive Order, which set an aim to reduce the number of Americans developing ESKD by 25% by 2030 through improved efforts to prevent, detect, and slow the progression of kidney disease.^35^ With the advent of novel therapies reported to slow CKD progression and prevent CKD complications, such as sodium-glucose cotransporter 2 inhibitors^36,37,38,39^ and nonsteroidal mineralocorticoid antagonists,^40^ disseminating these therapies consistently and equitably can benefit patients and may reduce disparities. However, it is concerning that recent data have already demonstrated race and ethnicity, sex, and socioeconomic inequities in the adoption of sodium-glucose cotransporter 2 inhibitors.^41^ Indeed, the delivery of equitable, high-quality chronic disease care may be worsened by the COVID-19 pandemic, and future studies should examine how the pandemic has affected care delivery and outcomes as a result of missed or deferred care.

### Limitations

Race and ethnicity derived from EHR records may not always represent self-reported race and do not capture multiracial or multiethnic status. We excluded patients without race or ethnicity information, which may result in bias if race or ethnicity is not missing at random. Our study population consisted of commercially insured and Medicare Advantage patients with continuous enrollment for at least a 1-year period before and after the index date, which strengthened ascertainment of clinical care delivery but also imposes a selection of patients who survived and did not change insurance. Notably, the racial and ethnic distribution of patients in this study population includes a greater prevalence of White and a lower prevalence of Asian, Black, and Hispanic patients compared with the general population.^42^ Accordingly, the findings observed should not be taken to be representative of racial and ethnic inequities in the general population, with a full spectrum of public and private insurance. We were unable to account for the use of nonprescription (over-the-counter) NSAID medications. We were unable to determine whether medications were prescribed because of CKD or an alternate clinical indication. Furthermore, we did not have detailed socioeconomic or lifestyle data because racial and ethnic differences in these factors may contribute to explaining the observed process and outcome measures.

## Conclusions

We assessed CKD care delivery in a large population of commercially insured and Medicare Advantage patients with CKD. We found that performance across the evaluated measures was similar or higher compared with nationally representative data but did not show substantive improvement from 2012-2013 to 2018-2019, suggesting a lack of progress in CKD care delivery for patients with CKD that underscores a continuing opportunity for implementation and dissemination strategies focused on improving CKD care delivery. In addition, compared with White patients, Asian, Black, and Hispanic patients were more likely to receive guideline-concordant ACEi or ARB treatment, statin treatment, referral to a nephrologist, and albuminuria monitoring but were similarly or less likely to achieve blood pressure and diabetes control targets for slowing CKD progression. Thus, in this commercially insured and Medicare Advantage patient population, differences in care delivery are unlikely to fully explain known racial and ethnic disparities in CKD progression and ESKD, and improvement of the processes of health care delivery alone may be inadequate for reducing these disparities.

## Notes

Supplement 1. **eTable 1.** Drug Classification Lists **eTable 2.** Diagnosis and Procedure Codes **eTable 3.** Study Population Characteristics by Year (N = 452 238) **eFigure 1.** Creatinine and UACR Testing Among Patients With Risk Factors for CKD **eFigure 2.** Study Flow Diagram **eFigure 3.** CKD Care Delivery Stratified by Race and Ethnicity and Diabetes Status **eFigure 4.** CKD Care Delivery Stratified by Race and Ethnicity and Age

Supplement 2. **Nonauthor Collaborators.** Centers for Disease Control and Prevention Chronic Kidney Disease Surveillance Team

## References

[1] United States Renal Data System. 2020 USRDS Annual Data Report: Epidemiology of Kidney Disease in the United States. National Institutes of Health, National Institute of Diabetes and Digestive and Kidney Diseases; 2020.

[2] Thompson S, James M, Wiebe N, ; Alberta Kidney Disease Network. Cause of death in patients with reduced kidney function. J Am Soc Nephrol. 2015;26(10):2504-2511. doi:10.1681/ASN.2014070714 25733525 PMC4587695

[3] Shlipak MG, Tummalapalli SL, Boulware LE, . The case for early identification and intervention of chronic kidney disease: conclusions from a Kidney Disease: Improving Global Outcomes (KDIGO) controversies conference. Kidney Int. 2021;99(1):34-47. doi:10.1016/j.kint.2020.10.012 33127436

[4] Nicholas SB, Kalantar-Zadeh K, Norris KC. Racial disparities in kidney disease outcomes. Semin Nephrol. 2013;33(5):409-415. doi:10.1016/j.semnephrol.2013.07.002 24119846 PMC3983362

[5] Crews DC, Bello AK, Saadi G; World Kidney Day Steering Committee. Burden, access, and disparities in kidney disease. Kidney Int Rep. 2019;4(3):372-379. doi:10.1016/j.ekir.2019.01.011 30899864 PMC6409385

[6] Albertus P, Morgenstern H, Robinson B, Saran R. Risk of ESRD in the United States. Am J Kidney Dis. 2016;68(6):862-872. doi:10.1053/j.ajkd.2016.05.030 27578184 PMC5123906

[7] Hounkpatin HO, Fraser SDS, Honney R, Dreyer G, Brettle A, Roderick PJ. Ethnic minority disparities in progression and mortality of pre-dialysis chronic kidney disease: a systematic scoping review. BMC Nephrol. 2020;21(1):217. doi:10.1186/s12882-020-01852-3 32517714 PMC7282112

[8] KDIGO CKD Work Group. KDIGO 2012 clinical practice guideline for the evaluation and management of chronic kidney disease. Kidney Int Suppl. 2013;3(1):5-14. 10.1038/ki.2013.243 23989362

[9] Kidney Disease: Improving Global Outcomes (KDIGO) Blood Pressure Work Group. KDIGO clinical practice guideline for the management of blood pressure in chronic kidney disease. Kidney Int Suppl. 2012;2(5):337-414. doi:10.1038/kisup.2012.46

[10] Kidney Disease: Improving Global Outcomes Lipid Workgroup. KDIGO clinical practice guideline for lipid management in chronic kidney disease. Kidney Int Suppl. 2013;3(3):259-305. doi:10.1038/kisup.2013.34

[11] James PA, Oparil S, Carter BL, . 2014 evidence-based guideline for the management of high blood pressure in adults: report from the panel members appointed to the Eighth Joint National Committee (JNC 8). JAMA. 2014;311(5):507-520. doi:10.1001/jama.2013.284427 24352797

[12] Whelton PK, Carey RM, Aronow WS, . 2017 ACC/AHA/AAPA/ABC/ACPM/AGS/APhA/ASH/ASPC/NMA/PCNA guideline for the prevention, detection, evaluation, and management of high blood pressure in adults: a report of the American College of Cardiology/American Heart Association Task Force on Clinical Practice Guidelines. Hypertension. 2018;71(6):e13-e115. doi:10.1161/HYP.0000000000000065 29133356

[13] American Diabetes Association. Summary of Revisions: *Standards of Medical Care in Diabetes—2021*. Diabetes Care. 2021;44(suppl 1):S4-S6. doi:10.2337/dc21-Srev 33298411

[14] Mefford MT, Rosenson RS, Deng L, . Trends in statin use among US adults with chronic kidney disease, 1999-2014. J Am Heart Assoc. 2019;8(2):e010640. doi:10.1161/JAHA.118.010640 30651020 PMC6497356

[15] Murphy DP, Drawz PE, Foley RN. Trends in angiotensin-converting enzyme inhibitor and angiotensin II receptor blocker use among those with impaired kidney function in the United States. J Am Soc Nephrol. 2019;30(7):1314-1321. doi:10.1681/ASN.2018100971 31167823 PMC6622408

[16] Chu CD, Powe NR, McCulloch CE, ; Centers for Disease Control and Prevention Chronic Kidney Disease Surveillance Team. Angiotensin-converting enzyme inhibitor or angiotensin receptor blocker use among hypertensive US adults with albuminuria. Hypertension. 2021;77(1):94-102. doi:10.1161/HYPERTENSIONAHA.120.16281 33190561 PMC7725867

[17] Wallace PJ, Shah ND, Dennen T, Bleicher PA, Crown WH. Optum Labs: building a novel node in the learning health care system. Health Aff (Millwood). 2014;33(7):1187-1194. doi:10.1377/hlthaff.2014.0038 25006145

[18] von Elm E, Altman DG, Egger M, Pocock SJ, Gøtzsche PC, Vandenbroucke JP; STROBE Initiative. The Strengthening the Reporting of Observational Studies in Epidemiology (STROBE) statement: guidelines for reporting observational studies. J Clin Epidemiol. 2008;61(4):344-349. doi:10.1016/j.jclinepi.2007.11.008 18313558

[19] Levey AS, Stevens LA, Schmid CH, ; CKD-EPI (Chronic Kidney Disease Epidemiology Collaboration). A new equation to estimate glomerular filtration rate. Ann Intern Med. 2009;150(9):604-612. doi:10.7326/0003-4819-150-9-200905050-00006 19414839 PMC2763564

[20] Taneja C, Berger A, Inglese GW, . Can dialysis patients be accurately identified using healthcare claims data? Perit Dial Int. 2014;34(6):643-651. doi:10.3747/pdi.2012.00328 24497600 PMC4164409

[21] Lee J, Chu C, Guzman D, . Albuminuria testing by race and ethnicity among patients with hypertension with and without diabetes. Am J Nephrol. 2019;50(1):48-54. doi:10.1159/000500706 31167180 PMC6620121

[22] Centers for Disease Control and Prevention. Chronic Kidney Disease (CKD) Surveillance System—United States. Published 2020. Accessed November 21, 2020. https://www.cdc.gov/ckd

[23] Gao SW, Oliver DK, Das N, . Assessment of racial disparities in chronic kidney disease stage 3 and 4 care in the Department of Defense health system. Clin J Am Soc Nephrol. 2008;3(2):442-449. doi:10.2215/CJN.03940907 18199843 PMC2390939

[24] Diamantidis CJ, Hale SL, Wang V, Smith VA, Scholle SH, Maciejewski ML. Lab-based and diagnosis-based chronic kidney disease recognition and staging concordance. BMC Nephrol. 2019;20(1):357. doi:10.1186/s12882-019-1551-3 31521124 PMC6744668

[25] Jolly SE, Navaneethan SD, Schold JD, . Chronic kidney disease in an electronic health record problem list: quality of care, ESRD, and mortality. Am J Nephrol. 2014;39(4):288-296. doi:10.1159/000360306 24714513 PMC4056768

[26] Yoon SSS, Carroll MD, Fryar CD. Hypertension prevalence and control among adults: United States, 2011-2014. NCHS Data Brief. 2015;(220):1-8. 26633197

[27] Rodríguez JE, Campbell KM. Racial and ethnic disparities in prevalence and care of patients with type 2 diabetes. Clin Diabetes. 2017;35(1):66-70. doi:10.2337/cd15-0048 28144049 PMC5241767

[28] Whelton PK. The elusiveness of population-wide high blood pressure control. Annu Rev Public Health. 2015;36(1):109-130. doi:10.1146/annurev-publhealth-031914-122949 25594330

[29] Whelton PK, Einhorn PT, Muntner P, ; National Heart, Lung, and Blood Institute Working Group on Research Needs to Improve Hypertension Treatment and Control in African Americans. Research needs to improve hypertension treatment and control in African Americans. Hypertension. 2016;68(5):1066-1072. doi:10.1161/HYPERTENSIONAHA.116.07905 27620388 PMC5063700

[30] Suarez J, Cohen JB, Potluri V, . Racial disparities in nephrology consultation and disease progression among veterans with CKD: an observational cohort study. J Am Soc Nephrol. 2018;29(10):2563-2573. doi:10.1681/ASN.2018040344 30120108 PMC6171274

[31] Peralta CA, Bibbins-Domingo K, Vittinghoff E, . APOL1 genotype and race differences in incident albuminuria and renal function decline. J Am Soc Nephrol. 2016;27(3):887-893. doi:10.1681/ASN.2015020124 26180129 PMC4769203

[32] Limou S, Nelson GW, Kopp JB, Winkler CA. APOL1 kidney risk alleles: population genetics and disease associations. Adv Chronic Kidney Dis. 2014;21(5):426-433. doi:10.1053/j.ackd.2014.06.005 25168832 PMC4157456

[33] Fowler KJ. Advancing American Kidney Health (AAKH): catalyst for investment in kidney diseases clinical trials and precision medicine: an opportunity to advance upstream interventions and the importance of nephrology. Clin J Am Soc Nephrol. 2020;15(12):1689-1691. doi:10.2215/CJN.03660320 32759179 PMC7769015

[34] Mendu ML, Tummalapalli SL, Lentine KL, . Measuring quality in kidney care: an evaluation of existing quality metrics and approach to facilitating improvements in care delivery. J Am Soc Nephrol. 2020;31(3):602-614. doi:10.1681/ASN.2019090869 32054692 PMC7062216

[35] Mehrotra R. Advancing American kidney health: an introduction. Clin J Am Soc Nephrol. 2019;14(12):1788. doi:10.2215/CJN.11840919 31690694 PMC6895493

[36] Perkovic V, Jardine MJ, Neal B, ; CREDENCE Trial Investigators. Canagliflozin and renal outcomes in type 2 diabetes and nephropathy. N Engl J Med. 2019;380(24):2295-2306. doi:10.1056/NEJMoa1811744 30990260

[37] Neal B, Perkovic V, Mahaffey KW, ; CANVAS Program Collaborative Group. Canagliflozin and cardiovascular and renal events in type 2 diabetes. N Engl J Med. 2017;377(7):644-657. doi:10.1056/NEJMoa1611925 28605608

[38] Wanner C, Inzucchi SE, Lachin JM, ; EMPA-REG OUTCOME Investigators. Empagliflozin and progression of kidney disease in type 2 diabetes. N Engl J Med. 2016;375(4):323-334. doi:10.1056/NEJMoa1515920 27299675

[39] Heerspink HJL, Stefánsson BV, Correa-Rotter R, ; DAPA-CKD Trial Committees and Investigators. Dapagliflozin in patients with chronic kidney disease. N Engl J Med. 2020;383(15):1436-1446. doi:10.1056/NEJMoa2024816 32970396

[40] Bakris GL, Agarwal R, Anker SD, Effect of finerenone on chronic kidney disease outcomes in type 2 diabetes. *N Engl J Med*. 2020;383(23):2219-2229. doi:10.1056/NEJMoa2025845 33264825

[41] Eberly LA, Yang L, Eneanya ND, . Association of race/ethnicity, gender, and socioeconomic status with sodium-glucose cotransporter 2 inhibitor use among patients with diabetes in the US. JAMA Netw Open. 2021;4(4):e216139. doi:10.1001/jamanetworkopen.2021.6139 33856475 PMC8050743

[42] US Census Bureau. National population by characteristics: 2010-2019. 2020. Accessed November 24, 2020. https://www.census.gov/data/tables/time-series/demo/popest/2010s-national-detail.html

