# Supplementary material for: Trends in Chronic Kidney Disease Care in the US by Race and Ethnicity, 2012-2019
Source: JAMA Netw Open. 2021 Sep 27;4(9):e2127014. doi: 10.1001/jamanetworkopen.2021.27014 (PMC8477264; doi:10.1001/jamanetworkopen.2021.27014)
Supplement: Supplement 1. — eTable 1. Drug Classification Lists eTable 2. Diagnosis and Procedure Codes eTable 3. Study Population Characteristics by Year (N = 452 238) eFigure 1. Creatinine and UACR Testing Among Patients With Risk Factors for CKD eFigure 2. Study Flow Diagram eFigure 3. CKD Care Delivery Stratified by Race and Ethnicity and Diabetes Status eFigure 4. CKD Care Delivery Stratified by Race and Ethnicity and Age [file jamanetwopen-e2127014-s001.pdf]

Supplementary Online Content

Chu CD, Powe NR, McCulloch CE, et al; Centers for Disease Control and Prevention Chronic Kidney Disease Surveillance Team. Trends in chronic kidney disease care in the US by race and ethnicity, 2012-2019. *JAMA Netw Open*. 2021;4(9):e2127014.
doi:10.1001/jamanetworkopen.2021.27014

eTable 1. Drug Classification Lists
eTable 2. Diagnosis and Procedure Codes
eTable 3. Study Population Characteristics by Year (N = 452 238)
eFigure 1. Creatinine and UACR Testing Among Patients With Risk Factors for CKD
eFigure 2. Study Flow Diagram
eFigure 3. CKD Care Delivery Stratified by Race and Ethnicity and Diabetes Status
eFigure 4. CKD Care Delivery Stratified by Race and Ethnicity and Age

This supplementary material has been provided by the authors to give readers additional information about their work.

eTable 1. Drug Classification Lists

**Antihypertensives**	
Vasodilators	Hydralazine, Minoxidil
Sympatholytic	Clonidine, Methyldopa, Guanfacine, Clonidine/Chlorthalidone, Methyldopa/HCTZ
ACE inhibitors	Lisinopril, Enalapril, Benazepril, Ramipril, Quinapril, Fosinopril, Captopril, Trandolapril, Moexipril, Perindopril
ARB	Losartan, Valsartan, Olmesartan, Irbesartan, Telmisartan, Candesartan, Azilsartan, Eprosartan
ARB + CCB	Amlodipine/Valsartan, Amlodipine/Olmesartan, Telmisartan/Amlodipine
ARB + thiazide	Losartan/HCTZ, Valsartan/HCTZ, Olmesartan/HCTZ, Telmisartan/HCTZ, Irbesartan/HCTZ, Azilsartan/Chlorthalidone, Candesartan/HCTZ
ACE + thiazide	Lisinopril/HCTZ, Benazepril/HCTZ, Enalapril/HCTZ, Quinapril/HCTZ, Fosinopril/HCTZ, Captopril/HCTZ, Moexipril/HCTZ
ACE + CCB	Amlodipine/Benazepril, Trandolapril/Verapamil
Renin inhibitor	Aliskiren
Renin inhibitor + thiazide	Aliskiren/HCTZ
ARB + CCB + thiazide	Olmesartan/Amlodipine/HCTZ, Amlodipine/Valsartan/HCTZ
Renin inhibitor + CCB	Aliskiren/Amlodipine
Vasodilators, coronary	Isosorbide mononitrate, isosorbide dinitrate, nitroglycerin
Vasodilators, combination	Isosorbide dinitrate/Hydralazine
CCB	Amlodipine, Diltiazem, Nifedipine, Verapamil, Felodipine, Nisoldipine, Isradipine, Nicardipine, Nimodipine
Alpha/Beta-adrenergic blocking agents	Carvedilol, Labetalol
Beta blockers	Metoprolol, Atenolol, Nebivolol, Propranolol, Sotalol, Bisoprolol, Nadolol, Acebutolol, Pindolol, Betaxolol
Beta blocker + thiazide	Atenolol/Chlorthalidone, Bisoprolol/HCTZ, Metoprolol/HCTZ, Bisoprolol/HCTZ, Propranolol/HCTZ, Nadolol/Bendroflumethiazide
CCB + statin	Amlodipine/Atorvastatin
Thiazides	HCTZ, Chlorthalidone, Metolazone, Indapamide, Chlorothiazide, Methyclothiazide
K-sparing diuretics	Spironolactone, Eplerenone, Amiloride, Triamterene
K-sparing diuretics + other	Triamterene/HCTZ, Spironolactone/HCTZ, Amiloride/HCTZ
Loop diuretics	Furosemide, Torsemide, Bumetanide, Ethacrynic Acid
**Diabetes**	
DPP-4i + TZD	Alogliptin/Pioglitazone
SGLT2i	Canagliflozin, Empagliflozin, Dapagliflozin
DPP-4i + Biguanide	Sitagliptin/Metformin, Saxagliptin/Metformin, Linagliptin/Metformin, Alogliptin/Metformin
Insulins	Glargine, Lispro, Detemir, Aspart, NPH/Regular, NPL/Lispro, NPH, Regular, Aspart, Lispro, Glulisine, Degludec

© 2021 Chu CD et al. *JAMA Network Open.*

Amylin analog	Pramlintide
GLP-1 agonist	Liraglutide, Exenatide, Dulaglutide, Albiglutide
DPP-4i	Sitagliptin, Linagliptin, Saxagliptin, Alogliptin
Insulin-release stimulant	Glipizide, Glimepiride, Glyburide, Repaglinide, Nateglinide, Chlorpropamide, Tolazamide, Tolbutamide
Biguanide	Metformin
Alpha-Glucosidase inhibitors	Acarbose, Miglitol
TZD	Pioglitazone, Rosiglitazone
TZD + Sulfonylurea	Pioglitazone/Glimepiride
Insulin-release stimulant + Biguanide	Glipizide/Metformin, Glyburide/Metformin, Repaglinide/Metformin
TZD + Biguanide	Pioglitazone/Metformin
**Statins**	
Statins	Atorvastatin, Simvastatin, Pravastatin, Rosuvastatin, Lovastatin, Pitavastatin, Fluvastatin
Statin + Niacin	Niacin/Lovastatin, Niacin/Simvastatin
Statin + Other	Ezetimibe/Simvastatin
CCB + statin	Amlodipine/Atorvastatin
**NSAIDs**	
NSAIDs	Meloxicam, Ibuprofen, Naproxen, Diclofenac, Nabumetone, Etodolac, Indomethacin, Sulindac, Piroxicam, Oxaproxin, Ketorolac, Flurbiprofen, Ketoprofen, Meclofenamate, Fenoprofen, Tolmetin, Mefenamic acid
NSAID + PPI	Naproxen/Esomeprazole

Abbreviations: ACE = angiotensin converting enzyme; ARB = angiotensin II receptor blocker; CCB = calcium channel blocker; DPP-4i = dipeptidyl peptidase-4 inhibitor; GLP-1 = glucagon-like peptide-1; HCTZ = hydrochlorothiazide; NSAID = nonsteroidal anti-inflammatory drug; PPI = proton pump inhibitor; SGLT2i = sodium-glucose cotransporter 2 inhibitor; TZD = thiazolidinedione.

© 2021 Chu CD et al. *JAMA Network Open.*

eTable 2. Diagnosis and Procedure Codes

Condition	Codes
End-Stage Kidney Disease	CPT-4: 90935, 90937, 90945, 90947, 99512
Hyperlipidemia	ICD-9: 272<br>ICD-10: E78
Diabetes	ICD-9: 250, 790.2, 790.21, 790.22, 790.29, 648.8x, 648.0x, 791.5, 277.7<br>ICD-10: V18.0, V77.1, E08x, E09x, E10x, E11x, E13x
Hypertension	ICD-9: 401.x, 402.x, 403.x, 404.x, 405.x<br>ICD-10: I10.x, I11.x, I12.x, I13.x, I15.x, I16.x
Coronary Heart Disease	ICD-9: 410.x, 412.x<br>ICD-10: I21.x, I22.x, I25.2
Heart Failure	ICD-9: 398.91, 402.01, 402.11, 402.91, 404.01, 404.03, 404.11, 404.13, 404.91, 404.93, 428.x<br>ICD-10: I09.81, I11.0, I13.0, I13.2, I50
Cerebrovascular Disease	ICD-9: 430.x, 431.x, 432.x, 433.x1, 434.x1, 435.x, 436.x, 438.x<br>ICD-10: V12.54, G45.0, G45.1, G45.2, G45.8, G45.9, G46.x, I60.x, I61.x, I62.x, I63.x, I69.x, Z86.73

Abbreviations: CPT = Current Procedural Terminology, ICD = International Classification of Disease.

© 2021 Chu CD et al. *JAMA Network Open.*

eTable 3. Study Population Characteristics by Year (N = 452,238)

**Characteristic**[a]	**2012-2013**	**2014-2015**	**2016-2017**	**2018-2019**
n	65,136	94,387	133,358	159,357
**Demographics**				
Age, mean ± SD	72.0 ± 10.3	73.1 ± 10.3	74.3 ± 10.1	75.2 ± 10.0
Female, n (%)	37690 (57.9)	54913 (58.2)	77177 (57.8)	92369 (58.0)
Race and ethnicity, n (%)				
White	55340 (85.0)	79356 (84.1)	111653 (83.7)	132806 (83.3)
Black	7090 (10.9)	10420 (11.0)	14949 (11.2)	17511 (11.0)
Hispanic	1698 (2.6)	2780 (2.9)	4628 (3.5)	6434 (4.0)
Asian	1008 (1.5)	1831 (1.9)	2128 (1.6)	2606 (1.6)
Neighborhood Education[b], n (%)				
Less than High School	1447 (2.2)	1724 (1.8)	1821 (1.4)	1668 (1.1)
High School	37029 (57.0)	49893 (53.1)	66443 (50.1)	71299 (46.8)
Less than College	23562 (36.3)	37084 (39.4)	55870 (42.1)	67606 (44.4)
College or higher	2869 (4.4)	5317 (5.7)	8526 (6.4)	11771 (7.7)
Medicare Advantage, n (%)	49450 (75.9)	76531 (81.1)	114072 (85.5)	141471 (88.8)
**Comorbidities**				
Hypertension, n (%)	56268 (86.4)	80283 (85.1)	112649 (84.5)	131774 (82.7)
Systolic BP, mean ± SD	130 ± 18	131 ± 19	131 ± 18	131 ± 19
Diastolic BP, mean ± SD	72 ± 11	73 ± 11	73 ± 11	73 ± 11
Diabetes, n (%)	28087 (43.1)	38920 (41.2)	55645 (41.7)	65542 (41.1)
HbA1c (%), mean ± SD	6.8 ± 1.6	6.8 ± 1.7	6.7 ± 1.6	6.6 ± 1.4
eGFR, mean ± SD	52 ± 18	52 ± 18	52 ± 18	51 ± 17
eGFR (ml/min/1.73m^2), n (%)				
≥60	10021 (15.4)	13940 (14.8)	20498 (15.4)	21211 (13.3)
45-59	34270 (52.7)	50754 (53.8)	71746 (53.9)	86104 (54.2)
30-44	15758 (24.2)	22112 (23.4)	31175 (23.4)	39303 (24.7)
<30	4946 (7.6)	7500 (8.0)	9667 (7.3)	12282 (7.7)
UACR (mg/g), median [IQR]	18 [7, 55]	19 [7, 61]	21 [8, 69]	22 [8, 73]
UACR (mg/g), n (%)				
<30	14751 (63.0)	21097 (61.0)	29766 (58.4)	35434 (57.7)
30-299	6640 (28.3)	10324 (29.9)	16215 (31.8)	19826 (32.3)
300-999	1305 (5.6)	1942 (5.6)	3173 (6.2)	3818 (6.2)
≥1000	732 (3.1)	1199 (3.5)	1845 (3.6)	2310 (3.8)
Hyperlipidemia, n (%)	56189 (86.3)	80849 (85.7)	115089 (86.3)	139067 (87.3)
Congestive Heart Failure, n (%)	14092 (21.6)	20248 (21.5)	30976 (23.2)	39678 (24.9)
Coronary Heart Disease, n (%)	7281 (11.2)	10074 (10.7)	15097 (11.3)	19306 (12.1)
Cerebrovascular Disease, n (%)	10087 (15.5)	14516 (15.4)	20862 (15.6)	25159 (15.8)

[a] All comparisons have p <0.001 except female sex (p = 0.38) and cerebrovascular disease (p = 0.04).
[b] Represents median education level achieved among all residents 25 years and older within the specified census block group according to the American Community Survey.
SI conversion: To convert HbA1c (%) to proportion of total hemoglobin, multiple by 0.01.
Abbreviations: BP = blood pressure, eGFR = estimated glomerular filtration rate, HbA1c = hemoglobin A1c, IQR = interquartile range, SD = standard deviation, UACR = urine albumin/creatinine ratio.

© 2021 Chu CD et al. *JAMA Network Open.*

eFigure 1. Creatinine and UACR Testing among Patients with Risk Factors for CKD

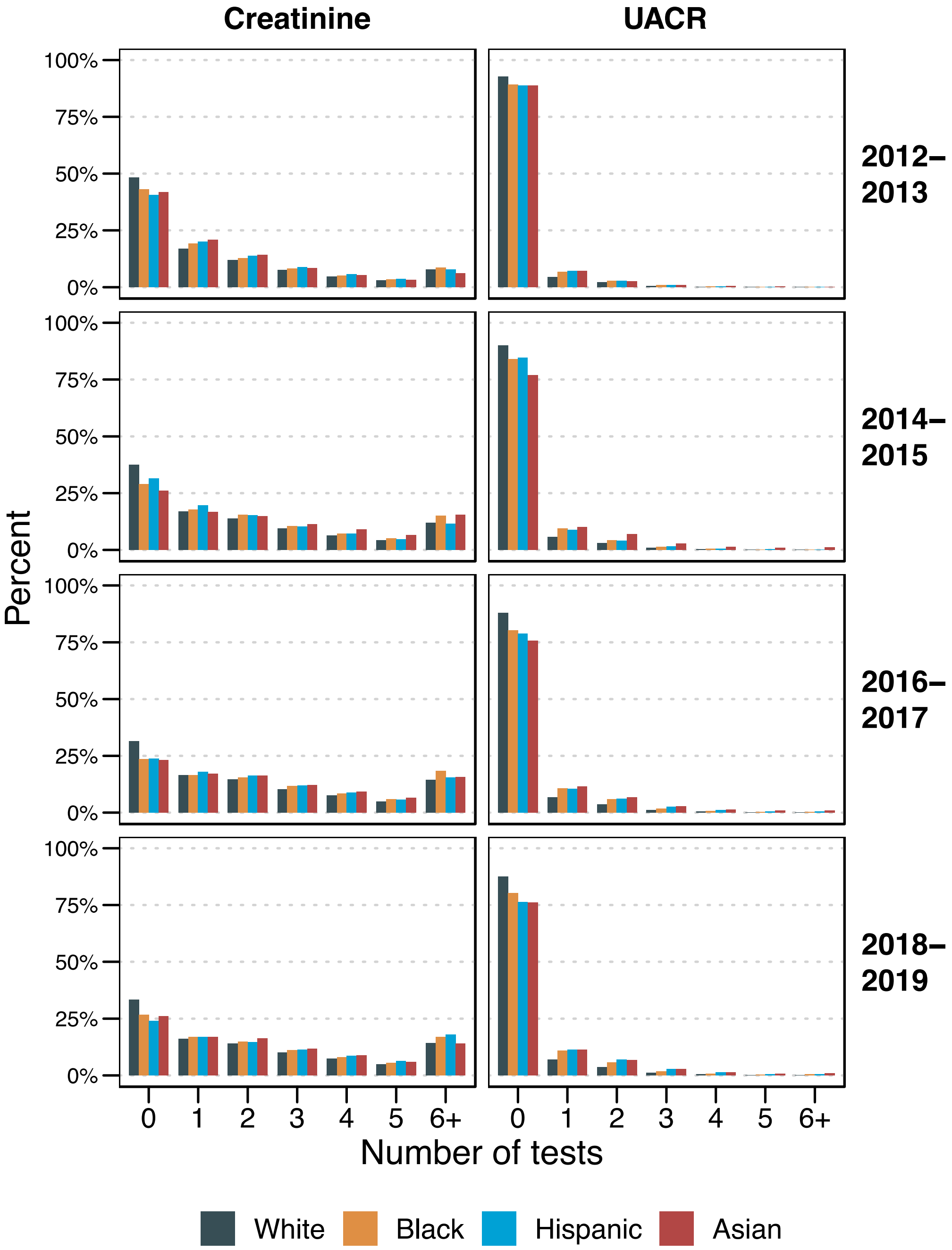

Each panel shows the percent of patients (by race and ethnicity) having either creatinine testing or UACR testing during a 2-year period of continuous enrollment. Patients with risk factors for CKD include those with hypertension, diabetes, or cardiovascular disease.
Abbreviations: CKD = chronic kidney disease, UACR = urine albumin/creatinine ratio.

© 2021 Chu CD et al. *JAMA Network Open.*

eFigure 2. Study Flow Diagram

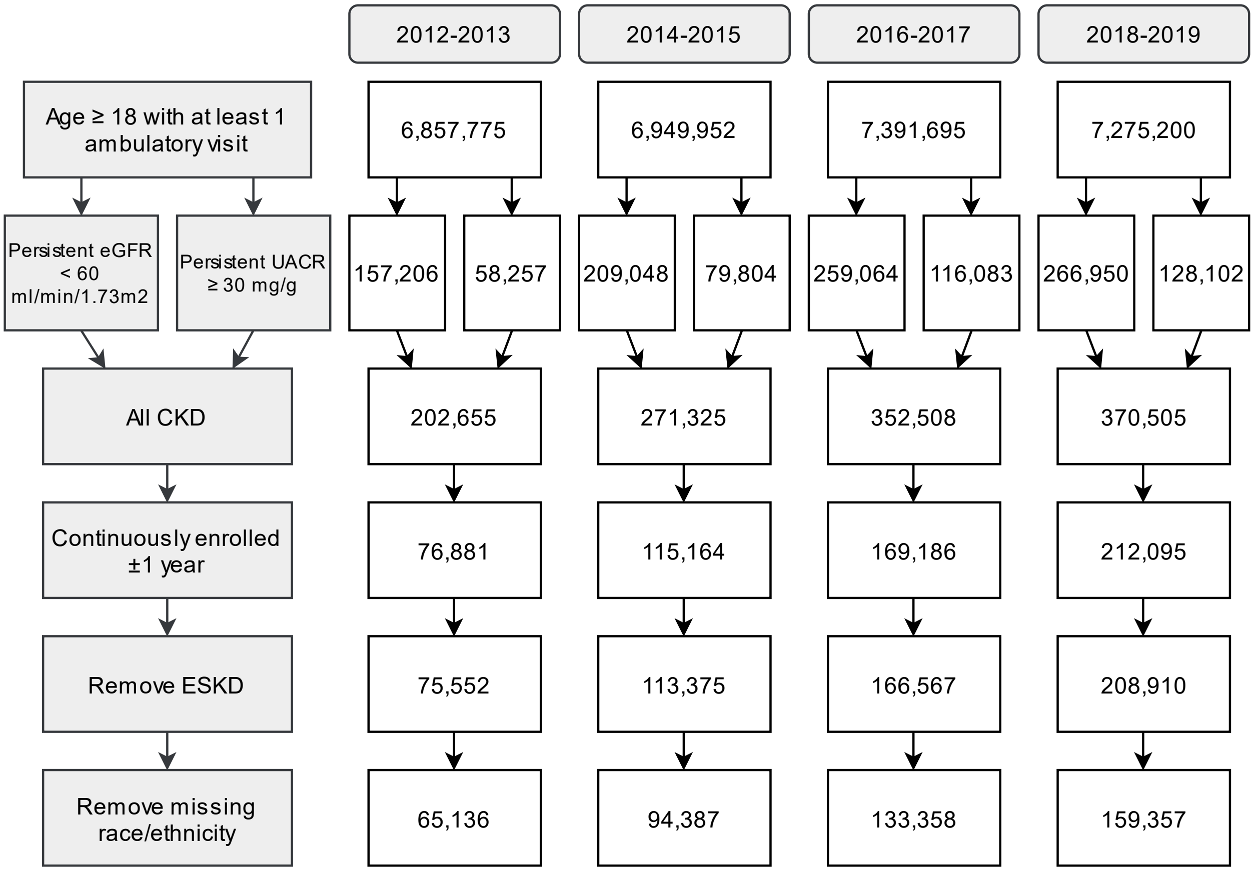

Abbreviations: CKD = chronic kidney disease, eGFR = estimated glomerular filtration rate, ESKD = end-stage kidney disease, UACR = urine albumin/creatinine ratio.

© 2021 Chu CD et al. *JAMA Network Open.*

eFigure 3. CKD Care Delivery Stratified by Race and Ethnicity and Diabetes Status

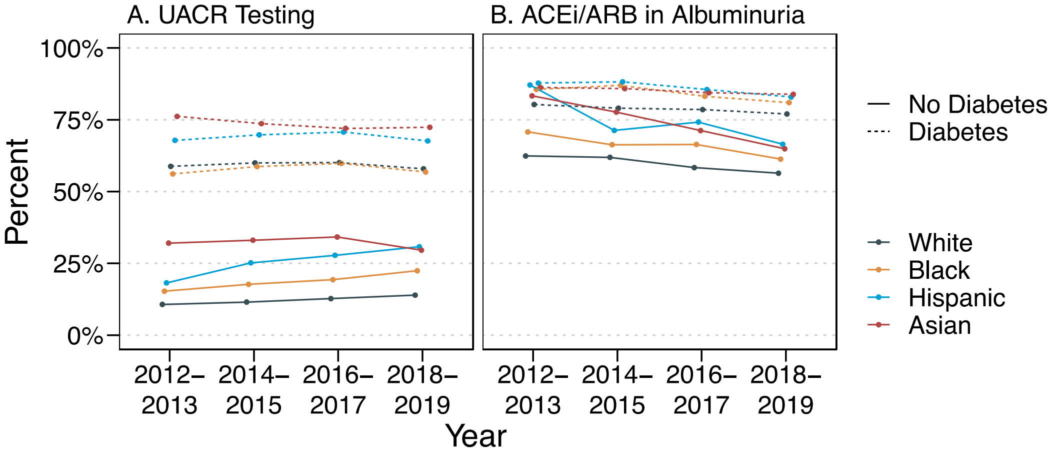

Abbreviations: ACEi = angiotensin converting enzyme inhibitor, ARB = angiotensin II receptor blocker, CKD = chronic kidney disease, UACR = urine albumin/creatinine ratio.

© 2021 Chu CD et al. *JAMA Network Open.*

eFigure 4: CKD Care Delivery Stratified by Race and Ethnicity and Age

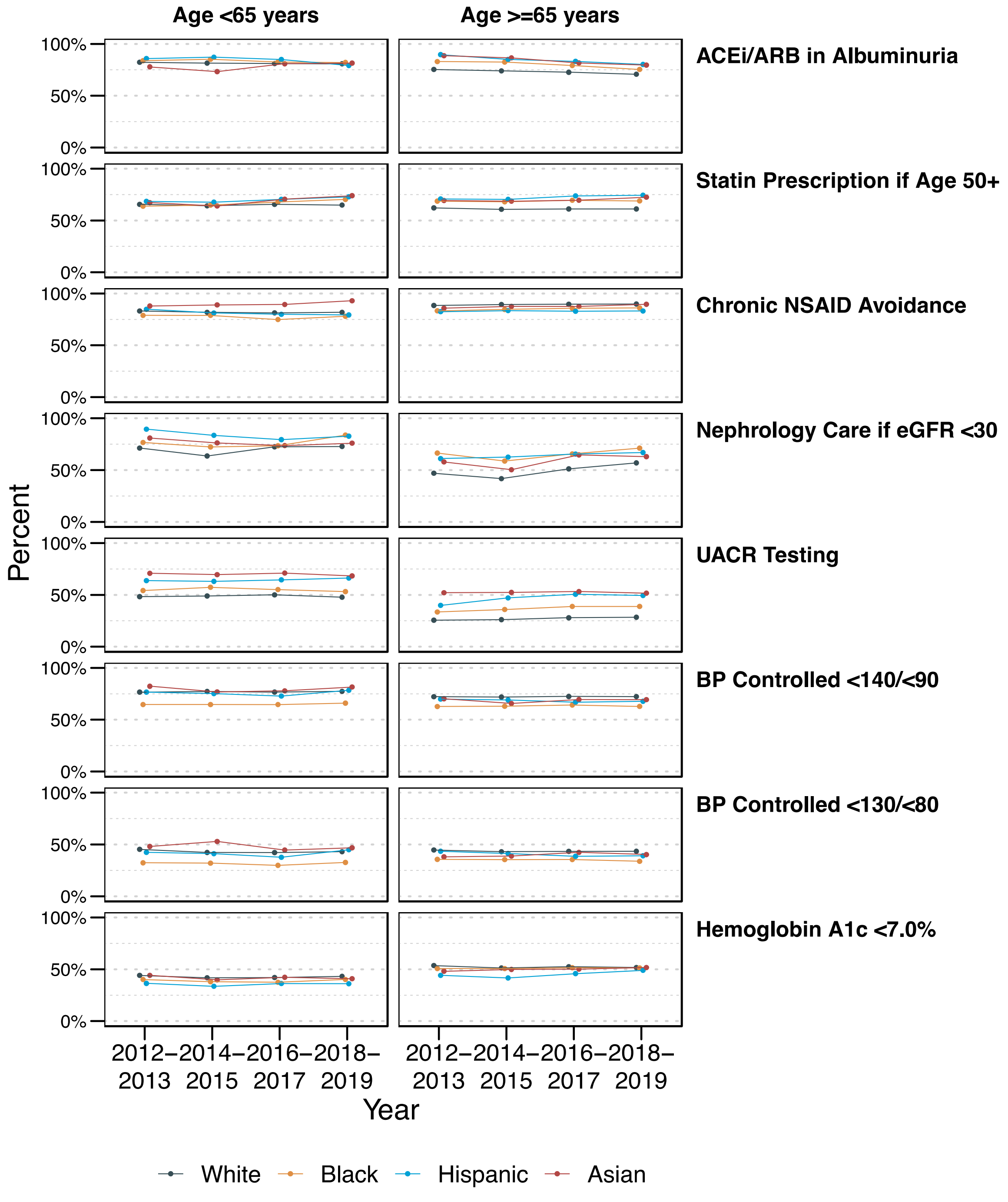

Abbreviations: ACEi = angiotensin converting enzyme inhibitor, ARB = angiotensin II receptor blocker, BP = blood pressure, CKD = chronic kidney disease, eGFR = estimated glomerular filtration rate, UACR = urine albumin/creatinine ratio.

© 2021 Chu CD et al. *JAMA Network Open.*
