# Supplementary material for: Trends in Chronic Kidney Disease Care in the US by Race and Ethnicity, 2012-2019
Source: JAMA Netw Open. 2021 Sep 27;4(9):e2127014. doi: 10.1001/jamanetworkopen.2021.27014 (PMC8477264; doi:10.1001/jamanetworkopen.2021.27014)
Supplement: Supplement 2. — Nonauthor Collaborators. Centers for Disease Control and Prevention Chronic Kidney Disease Surveillance Team [file jamanetwopen-e2127014-s002.pdf]

Supplemental Online Content: Nonauthor Collaborators

*Indicates required information. Only first name, last name, and suffix will appear in PubMed.

*Group Name(s): Centers for Disease Control and Prevention Chronic Kidney Disease Surveillance Team							
***First Name and Middle Initial(s)**	***Last Name**	***Suffix (eg, Jr, III)**	Academic Degrees	Institution	Location (city, state/province, country)	Role or Contribution, eg, chair, principal investigator	Group (if more than 1 Group listed in the byline) and/or Subgroup (eg, Steering Committee)
Neil	Powe		MD, MPH, MBA	University of California, San Francisco	San Francisco, CA	PI	
Rajiv	Saran		MD	University of Michigan	Ann Arbor, MI	PI	
Vahakn	Shahinian		MD	University of Michigan	Ann Arbor, MI		
Michael	Heung		MD	University of Michigan	Ann Arbor, MI		
Brenda	Gillespie		PhD	University of Michigan	Ann Arbor, MI		
Hal	Morgenstern		PhD	University of Michigan	Ann Arbor, MI		
William	Herman		MD, MPH	University of Michigan	Ann Arbor, MI		
Kara	Zivin		PhD	University of Michigan	Ann Arbor, MI		
Debbie	Gipson		MD, MS	University of Michigan	Ann Arbor, MI		
Zubin	Modi		MD	University of Michigan	Ann Arbor, MI		
Jennifer	Bragg-Gresham		PhD	University of Michigan	Ann Arbor, MI		
Diane	Steffick		PhD	University of Michigan	Ann Arbor, MI		
Yun	Han		PhD	University of Michigan	Ann Arbor, MI		
Xiaosong	Zhang		MA	University of Michigan	Ann Arbor, MI		
April	Wyncott		MPH	University of Michigan	Ann Arbor, MI		
Tanushree	Banerjee		PhD	University of California, San Francisco	San Francisco, CA		
Delphine	Tuot		MDCM, MAS	University of California, San Francisco	San Francisco, CA		
Chi	Chu		MD, MAS	University of California, San Francisco	San Francisco, CA		
Chi-yuan	Hsu		MD, MSc	University of California, San Francisco	San Francisco, CA		
Josef	Coresh		MD, PhD	Johns Hopkins University	Baltimore, MD		
Charles	McCulloch		PhD	University of California, San Francisco	San Francisco, CA		

Supplemental Online Content: Nonauthor Collaborators

*Indicates required information. Only first name, last name, and suffix will appear in PubMed.

***First Name and Middle Initial(s)**	***Last Name**	***Suffix (eg, Jr, III)**	Academic Degrees	Institution	Location (city, state/province, country)	Role or Contribution, eg, chair, principal investigator	Group (if more than 1 Group listed in the byline) and/or Subgroup (eg, Steering Committee)
Deidra	Crews		MD, ScM	Johns Hopkins University	Baltimore, MD		
Janet	Pella		BS	University of California, San Francisco	San Francisco, CA		
Nilka	Ríos Burrows		MPH	Centers for Disease Control and Prevention	Atlanta, GA		
Mark	Eberhardt		PhD	Centers for Disease Control and Prevention	Atlanta, GA		
Alain	Koyama		ScD	Centers for Disease Control and Prevention	Atlanta, GA		
Juanita	Mondesire			Centers for Disease Control and Prevention	Atlanta, GA		
Meda	Pavkov		MD, PhD	Centers for Disease Control and Prevention	Atlanta, GA		
Deborah	Rolka		MS	Centers for Disease Control and Prevention	Atlanta, GA		
Sharon	Saydah		PhD	Centers for Disease Control and Prevention	Atlanta, GA		
